# Estimating Lumbar Spine Foraminal Disc Measurements Using Ultrasound and X-Ray Imaging Through Advanced Image Annotation, Processing, and Mathematical Modeling During Chiropractic Traction Procedures: A Feasibility Study

**DOI:** 10.3390/bioengineering13030330

**Published:** 2026-03-12

**Authors:** Chandra Bhagi, Maruti Ram Gudavalli, Ralph A. Kruse, James M. Cox

**Affiliations:** 1 Research Department, College of Chiropractic Medicine, Keiser University, West Palm Beach, FL 33411, USA; bhagics@gmail.com (C.B.); rkruse@keiseruniversity.edu (R.A.K.); 2 Fort Wayne Chiropractic Radiological Center, Fort Wayne, IN 46845, USA

**Keywords:** chiropractic, generative artificial intelligence, radiograph, spinal measurements, ultrasound, X-ray

## Abstract

Accurate measurement of spinal metrics is critical for diagnosing and treating spinal disorders. However, discrepancies between X-ray and ultrasound imaging data pose a challenge in standardizing clinical assessments. This study introduces a novel methodology that combines geometric scaling factors and extrapolation techniques to align spinal metrics from X-ray and ultrasound modalities. Data were collected from fifteen healthy adult volunteers (8 males, 7 females) aged from early adulthood to middle age, all without a history of low back pain, who underwent a standardized chiropractic traction protocol. X-ray imaging was performed pre-procedure, and ultrasound imaging was conducted both pre-procedure and during the procedure at the L3–L4, L4–L5, and L5–S1 levels under graded traction forces (1.8 kg, 3.6 kg, 5.4 kg, and 11.3 kg). Extrapolation methods were applied to standardize measurements across pre- and during-procedure conditions. Significant findings include consistent increases in spinal metrics, such as height and area, indicating positive elongation and flexibility under progressive weights. The integration of these methods bridges the gap between static and real-time imaging data, potentially enhancing diagnostic accuracy and leads to clinical relevance. This proof-of-concept study lays the groundwork for developing standardized spinal imaging protocols and adapting the methodology to broader imaging applications for improved patient outcomes.

## 1. Introduction

Low back pain (LBP) remains one of the leading causes of disability worldwide, highlighting the critical need for accurate diagnostic and treatment methodologies [1]. Numerous studies underscore the growing burden of LBP and the need for innovative diagnostic approaches [1,2,3]. The global socioeconomic impact of LBP is significant, highlighting its widespread prevalence and chronic nature [1,2,3]. Manipulative procedures have been used for treating LBP and are recommended for its treatment [4]. Chiropractors typically assess and treat patients with LBP using spinal manipulative therapy, which includes a specific technique known as the flexion distraction. The force-based flexion-distraction technique is a table-assisted, low-velocity, variable-amplitude form of spinal manipulation. The intervertebral foramen (IVF) and disc height measurements are pivotal in diagnosing and managing lumbar spine disorders [5,6]. Studies have shown the biomechanical effects of the flexion-distraction technique on the lumbar spine, including increases in intervertebral disc heights and IVF area [7,8]. Accurate imaging techniques play an important role in treating LBP [9]. Studies are exploring the clinical impact of emerging imaging technologies, reinforcing the necessity for standardized measurement protocols to improve diagnostic accuracy and treatment outcomes [10,11,12].

Clinicians have access to a variety of imaging modalities for evaluating LBP. Traditionally, conventional radiography has been the gold standard for these measurements; however, its static nature limits its effectiveness in capturing dynamic biomechanical changes [13]. In addition, conventional radiography, also known as X-ray imaging, involves radiation exposure and mainly visualizes the bony structures of the lower back [14]. Other imaging modalities such as computed tomography (CT) and magnetic resonance imaging (MRI) are widely used due to their high-resolution. However, CT is associated with higher radiation exposure and cost compared with conventional radiography, while MRI is costlier than radiography or CT [15,16]. The limitations associated with these imaging techniques have necessitated the exploration of alternatives, such as ultrasound, which is non-ionizing, cost effective, and provides real-time insights into spinal mechanics [17]. Moreover, ultrasound has been found to reliably measure the distance between lumbar interspinous processes [18].

Despite their advantages, current imaging methods have notable limitations [13]. Conversely, ultrasound imaging offers real-time visualization of soft tissue and intervertebral dynamics [19]; however, the lack of standardized measurement protocols often leads to inconsistencies. Additionally, a robust computational framework to align and integrate these two modalities for precise measurement comparisons remains largely unexplored.

The primary aim of this study was to estimate intervertebral foramen (IVF) height, width, and area, as well as posterior disc height (PDH) and anterior disc height (ADH), by integrating ultrasound-derived dynamic responses under graded traction with static X-ray-based measurements. The secondary aim was to employ Generative Artificial Intelligence (GenAI) solely for post-analysis interpretation of finalized results, without involvement in measurement, scaling, or statistical computation.

## 2. Materials and Methods

### 2.1. Methodology Overview and Workflow

**Figure 1:**
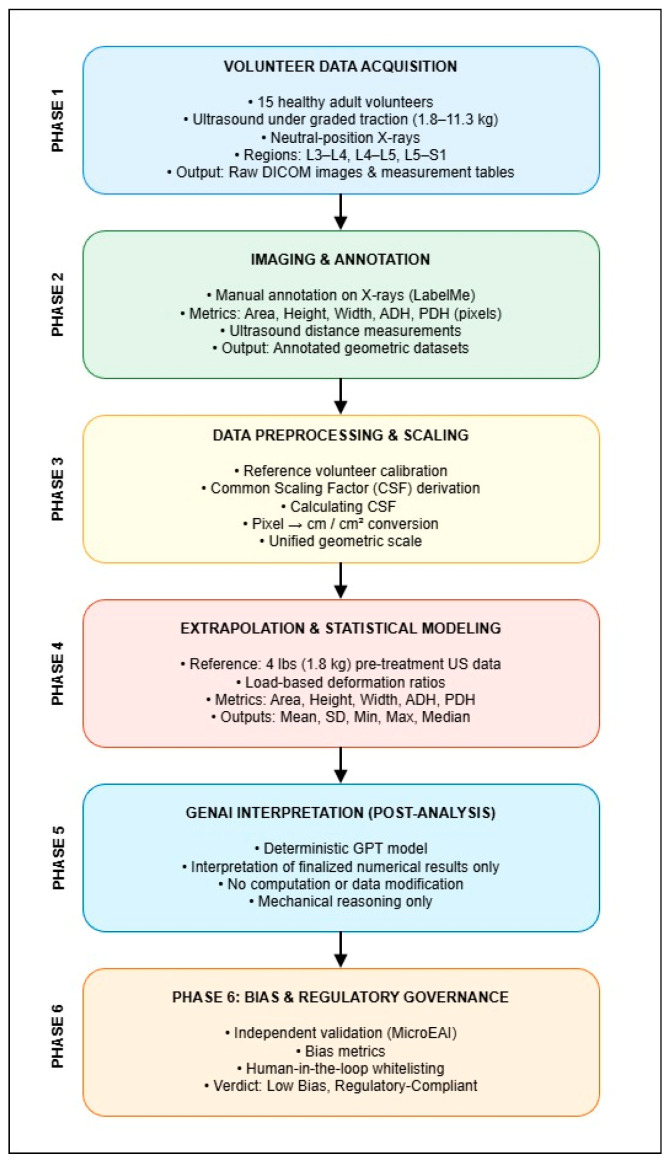
End-to-end analytical workflow for multimodal spinal measurement analysis integrating X-ray and ultrasound imaging. The workflow illustrates the sequential phases of data acquisition, annotation, geometric scaling, extrapolation and statistical modeling, AI-assisted interpretation, and bias governance.

To ensure methodological transparency and clearly distinguish deterministic numerical computation from artificial intelligence-assisted interpretation, an end-to-end analytical workflow was defined, as illustrated in Figure 1. The workflow outlines the complete processing pipeline, beginning with volunteer data acquisition and image annotation and progressing through geometric scaling, extrapolation, statistical analysis, and post-analysis interpretation.

The methodology was organized into sequential phases, each with a clearly defined role. Image acquisition, annotation, geometric scaling, extrapolation, and statistical calculations were performed using deterministic, annotation-driven mathematical techniques. Generative Artificial Intelligence (OpenAI, San Francisco, CA 94110, USA) was not involved in measurement, scaling, extrapolation, or statistical computation. Its role was strictly limited to post-analysis interpretation of finalized numerical results under deterministic and bias-governed constraints.

The overall analytical workflow consists of six phases, summarized below:

1. Volunteer Data Acquisition—Collection of baseline X-ray images (Model # VSW2556RE3, General Electric, Chicago, IL, USA) and Ultrasound images (GE Logiq P9, General Electric, Chicago, IL, USA) measurements under graded traction loads from healthy volunteers.
2. Imaging and Annotation—Manual annotation of X-ray and ultrasound images to identify anatomical landmarks and extract geometric measurements.
3. Data Preprocessing and Geometric Scaling—Application of a Common Scaling Factor (CSF) to standardize measurements across imaging modalities.
4. Extrapolation and Statistical Modeling—Calculation of extrapolated spinal metrics and descriptive statistical summaries across traction loads.
5. GenAI Interpretation (Post-Analysis)—Use of a generative AI model to provide structured descriptive interpretations of finalized numerical results.
6. Bias and Regulatory Governance—Independent validation of AI-generated interpretations using bias metrics and human-in-the-loop review.

This structured workflow maintains a clear separation between biomechanical data This structured workflow ensures a clear separation between biomechanical data generation and AI-assisted interpretation, supporting reproducibility, regulatory alignment, and clinical interpretability.

### 2.2. Setting, Participants, Imaging & Procedure

The study was conducted from February to June 2022 at the Keiser University College of Chiropractic Medicine in West Palm Beach, Florida.

#### 2.2.1. Participants & Criteria

Inclusion criteria required generally healthy adult volunteers (≥18 years) with no history of low back pain who could comfortably lie in a prone position. Exclusion criteria included pregnancy and any condition preventing participants from remaining prone for at least five minutes.

A total of 15 healthy adult volunteers (8 males, 7 females) were recruited through announcements at Keiser University. Mean (SD) age was 36 (14) years; mean (SD) BMI was 23.2 (2.4) kg/m^2^; mean (SD) height was 169.8 (7.4) cm; and mean (SD) weight was 67.3 (10.4) kg. All participants reported no prior history of low back pain (LBP). Detailed demographic characteristics are provided in the Supplementary Material.

#### 2.2.2. Imaging & Procedure

All volunteers were positioned prone on a specialized flexion-distraction table and underwent a standardized flexion-distraction procedure performed by an experienced, licensed clinician. Imaging was conducted under pre-procedure and during-procedure conditions at the L3–L4, L4–L5, and L5–S1 levels using graded traction forces of 1.8 kg, 3.6 kg, 5.4 kg, and 11.3 kg.

#### 2.2.3. X-Ray Imaging

X-ray imaging was performed by a certified radiologic technologist. Volunteers were positioned in a neutral prone posture. Baseline X-ray images were acquired prior to traction and served as the static anatomical reference for all subsequent measurements. X-ray imaging was performed only under pre-procedure conditions.

#### 2.2.4. Ultrasound Imaging

Ultrasound imaging was performed by a registered sonographer certified by the American Registry for Diagnostic Medical Ultrasound. Imaging was conducted using a curvilinear 2–5 MHz transducer (GE Logiq P9, General Electric, Chicago, IL, USA). The transducer was positioned perpendicular to the spine at predefined lumbar levels to capture interspinous distances under graded traction loads. Ultrasound imaging was used to assess structural changes during the procedure.

### 2.3. Annotation

Image annotation was used to establish baseline spinal measurements that served as reference values for extrapolation. Advanced annotation tools were applied to identify intervertebral foramen (IVF) regions and quantify key metrics, including area, height, width, posterior disc height (PDH), and anterior disc height (ADH). These baseline values formed the reference framework for subsequent analyses (Appendix A).

All annotations for both X-ray and ultrasound images were performed manually by a Diplomate of the American Board of Chiropractic Radiology. Regions of interest were delineated using an annotation tool that generated precise coordinate data. These coordinates were subsequently used to compute spinal measurements through deterministic mathematical calculations.

#### 2.3.1. Annotation of X-Ray BASE Values

X-ray image annotation plays a fundamental role in establishing the baseline values for spinal measurements. Advanced image annotation tools were used to identify IVF regions and quantify critical metrics such as PDH, ADH, and overall disc height. These annotated values served as the “BASE values,” forming the reference framework for subsequent extrapolation processes (Appendix A).

The annotation process focused on accurately marking the IVF regions and disc heights for the key lumbar regions (L3–L4, L4–L5, L5–S1) for both of the volunteers. These base values are critical as they provide the initial high-resolution, static measurements from X-ray images, which are later extrapolated and aligned with real-time ultrasound data across varying traction conditions (Figure 1). This standardized approach ensured consistency, accuracy, and reliability, forming the foundation for scaling and extrapolation techniques to align spinal metrics across imaging modalities.

##### IVF Area Calculation

**Figure 2:**
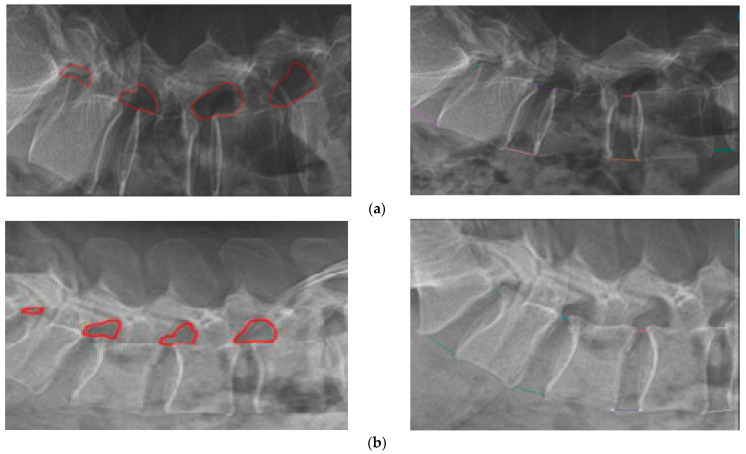
Image annotation of intervertebral foramen (IVF), posterior disc height (PDH), and anterior disc height (ADH) using lumbar X-ray images. Example of the image annotation and quantification of the IVF, PDH, and ADH in lumbar X-ray images. (**a**) The X-ray images of Volunteer A. (**b**) The X-ray images of Volunteer B.

The radiologist manually annotated the IVF regions (Figure 2) using an annotation tool that generated JSON coordinates defining the boundaries of the IVF. To determine the enclosed area, the polygon area formula (Shoelace Theorem) was applied to the extracted coordinates (Formula 2). This approach enabled precise calculation of the IVF region, which served as the baseline reference measurement. The calculated IVF area was subsequently used for extrapolation under graded traction conditions in ultrasound imaging.

##### PDH and ADH Calculations

Unlike IVF area measurements, posterior disc height (PDH) and anterior disc height (ADH) were annotated using an annotation tool that generated JSON coordinates corresponding to lumbar tip landmarks. PDH was calculated as the distance between posterior lumbar tip points, and ADH as the distance between anterior lumbar tip points (Formula 3). These values were recorded across the L3–L4, L4–L5, and L5–S1 regions during the pre-procedure phase, establishing baseline disc height measurements for subsequent extrapolation and comparison across graded traction loads.

#### 2.3.2. Annotation of Ultrasound Images

A standardized annotation and measurement protocol was developed for ultrasound images, focusing on the measurement of distances between lumbar spinous processes. This approach enabled accurate and consistent quantification of lumbar spinal metrics and provided dynamic insights into spinal changes during traction. Using this method, ultrasound data captured real-time spinal responses to graded mechanical loads, complementing static measurements derived from X-ray images.

**Figure 3:**
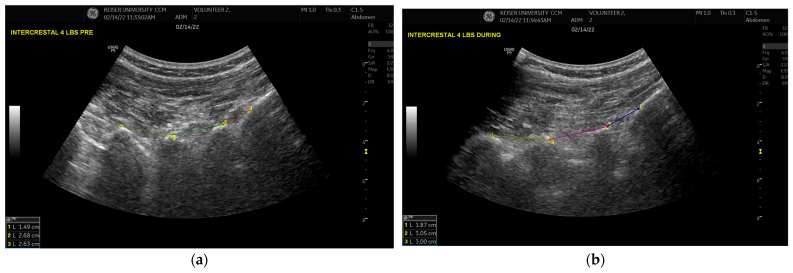
Ultrasound image annotation of lumbar regions. Example of annotation in Volunteer 2: (**a**) image obtained pre-procedure (1.8 kg traction force); (**b**) image obtained during-procedure (1.8 kg traction force).

Annotated lumbar distances (Formula 2) were calculated from JSON coordinates generated by the annotation tool. These measurements were subsequently integrated into the scaling and extrapolation framework to align ultrasound-derived metrics with X-ray baseline values (Section 2.5). Lumbar distances were recorded at 1.8 kg, 3.6 kg, 5.4 kg, and 11.3 kg under both pre-procedure and during-procedure conditions for the two volunteers (Figure 3).

### 2.4. Common Scaling Factor

To harmonize pixel-based X-ray measurements with physical ultrasound measurements, a single Common Scaling Factor (CSF) was derived. The CSF was calculated using matched anatomical landmarks from a reference volunteer by relating ultrasound-measured distances (in millimeters) to corresponding X-ray distances (in pixels).

This yielded a constant conversion factor (CSF = 0.518 cm/px), which was applied uniformly across all volunteers, lumbar regions, and measured variables. The CSF was used to convert X-ray baseline measurements from pixel units into physical dimensions (cm and cm^2^), ensuring a unified geometric scale across the dataset.

### 2.5. Extrapolation of Ultrasound Measurements

To ensure consistency and reliability across imaging modalities, extrapolation was performed using X-ray measurements as baseline reference values. Because X-ray images provide high-resolution, static anatomical references, the Common Scaling Factor (CSF) (Formula 6) was applied to standardize ultrasound-derived measurements, compensating for differences in image resolution, orientation, and pixel scaling.

The extrapolation process followed a structured framework in which five key metrics were derived by projecting X-ray baseline values across graded traction loads under both pre-procedure and during-procedure conditions:

(1) IVF Area Extrapolation

Ultrasound-derived distance measurements were adjusted using the CSF to estimate intervertebral foramen (IVF) area (Formula 4), enabling alignment with X-ray–defined anatomical dimensions.

(2) & (3) IVF Width & IVF Height Adjustments Using CSF

Due to differences in pixel resolutions between X-ray and ultrasound images, CSF was applied to extrapolate both width and height values, ensuring proper anatomical scaling (Formula 6).

The CSF was computed as a geometric mean–based scaling transformation that integrates horizontal and vertical adjustments to achieve standardized measurements across imaging modalities. This allowed the ultrasound-based IVF width and height measurements to be accurately mapped onto X-ray-based anatomical dimensions.

(4) & (5) Posterior Disc Height (PDH) & Anterior Disc Height (ADH) Extrapolation

PDH and ADH measurements from ultrasound images were standardized using the Common Scaling Factor (CSF) to ensure consistency in disc height estimation across imaging modalities (Formula 5). This transformation minimized discrepancies caused by variations in image resolution, probe positioning, and compression effects.

After conversion into physical units using the CSF, X-ray baseline measurements served as the anatomical reference for extrapolation. Ultrasound-derived measurements under graded traction were used to compute load-specific deformation ratios, with the 4 lbs (1.8 kg) pre-procedure condition selected as the reference baseline.

For each volunteer, lumbar region, and metric (Area, Height, Width, ADH, PDH), extrapolated values were calculated as:

$$\mathrm{Extrapolated}\;\mathrm{Metric}\;=\;\text{X-ray}\;\mathrm{BASE}\;\mathrm{Value}\;\times \;\left(\frac{\mathrm{Ultrasound}\;\mathrm{Measurement}\;\mathrm{at}\;\mathrm{Load}}{\mathrm{Ultrasound}\;\mathrm{Measurement}\;\mathrm{at}\;4\;\mathrm{lbs}\;\mathrm{Pre}\text{-}\mathrm{procedure}}\right)$$

This computation was performed independently across all traction loads and procedure conditions. Statistical summaries—including mean, standard deviation, minimum, maximum, and median—were then calculated across volunteers for each lumbar region and load level.

Why Was this Approach Taken?

- X-ray imaging serves as the anatomical “gold standard” with stable, well-defined, and consistent measurements, making it the ideal baseline for extrapolation.
- Ultrasound imaging, though dynamic, is prone to distortions due to variations in probe placement, angle, and pressure, necessitating a standardization process.
- By leveraging the CSF, we ensured that ultrasound-based metrics were reliably transformed into X-ray-defined standardized dimensions, enabling accurate clinical assessments.
- This extrapolation framework successfully standardized spinal measurements across imaging modalities, ensuring a robust, reproducible, and clinically valid methodology for future research.

## 3. Results

The data is based on 15 volunteers with Age: Mean and SD, Height, Weight, BMI, 7 Females and 8 Males mostly Caucasian

### 3.1. Pre-Distraction Baseline Measurements

Pre-distraction measurements were analyzed to establish baseline lumbar spine geometry prior to the application of traction forces. Mean values and standard deviations were calculated for anterior disc height (ADH), intervertebral foramen (IVF) area, overall height, posterior disc height (PDH), and width at the L3–L4, L4–L5, and L5–S1 levels.

Across all regions, IVF area demonstrated the greatest absolute magnitude and variability, reflecting inherent anatomical differences between lumbar levels. The L3–L4 region exhibited the largest mean baseline area and width values, followed by L4–L5 and L5–S1. Disc height measurements (ADH and PDH) showed comparatively lower variability across regions, indicating consistent baseline disc geometry among participants prior to distraction.

These pre-distraction baseline values provide a stable anatomical reference for subsequent extrapolation and deformation analysis, ensuring that observed changes during traction are interpreted relative to each region’s intrinsic geometric characteristics rather than inter-subject variability.

#### Pre-Distraction Regional Characteristics and Variability

**Figure 4:**
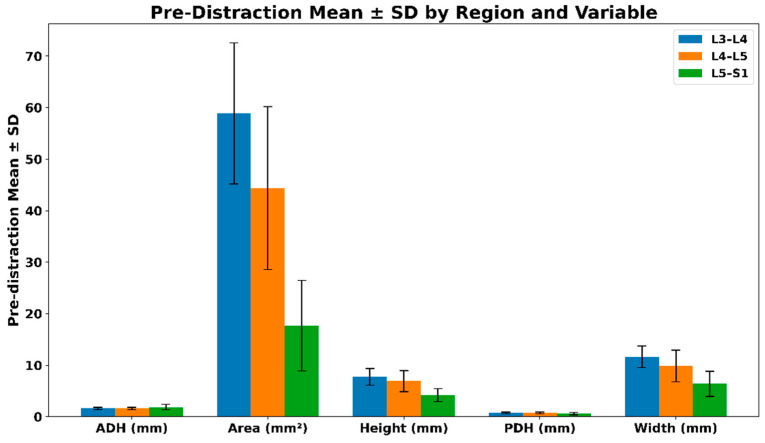
Pre-distraction mean ± SD values by lumbar region.

Figure 4 illustrates the pre-distraction mean ± standard deviation for each lumbar region, stratified by geometric variable. Region-specific differences were observed across all measured parameters. The L3–L4 and L4–L5 levels demonstrated larger baseline IVF area, height, and width compared with L5–S1, consistent with known anatomical tapering toward the lumbosacral junction. The pre-distraction mean ± SD values for the lumbar regions are summarized in Table 1.

### 3.2. Extrapolated Values During the Distraction Procedure

Extrapolated biomechanical values were analyzed to quantify lumbar deformation during the distraction procedure across graded traction loads. For each lumbar region and variable, delta values were computed as the difference between pre-distraction and during-distraction measurements at 4 lbs (1.8 kg), 8 lbs (3.6 kg), 12 lbs (5.4 kg), and 25 lbs (11.3 kg).

These delta values were derived from the extrapolated dataset for all measured variables, including intervertebral foramen area, height, width, anterior disc height (ADH), and posterior disc height (PDH).

For each load level and region, mean and standard deviation (SD) were calculated across the volunteer cohort to characterize the magnitude and variability of load-dependent structural responses. This approach enables systematic comparison of deformation patterns across loads and regions while accounting for inter-subject variability.

#### 3.2.1. IVF Area—Mean and SD of Extrapolated Values

**Figure 5:**
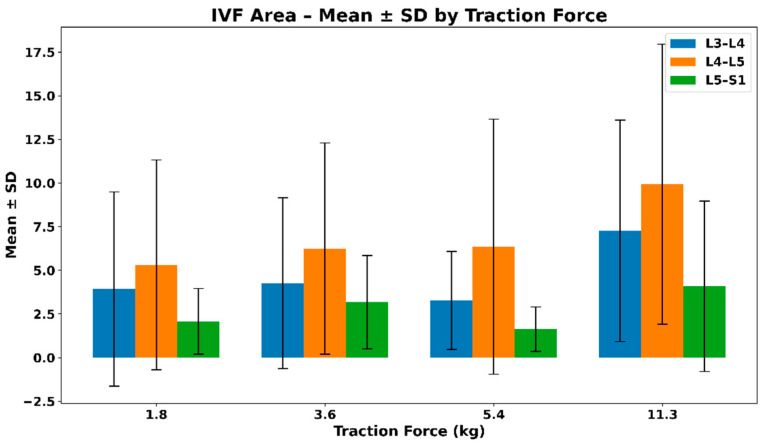
Mean ± SD change in intervertebral foramen (IVF) area across lumbar regions under graded distraction loads.

Figure 5 shows the mean ± SD change in intervertebral foramen area across lumbar regions under increasing distraction loads. All regions demonstrate positive mean area changes, with the largest absolute responses observed at higher loads, particularly at L4–L5. Increasing variability with load reflects inter-subject differences in biomechanical response rather than a uniform dose–response pattern. The mean ± SD values of the extrapolated intervertebral foramen (IVF) area across different traction loads are summarized in Table 2.

#### 3.2.2. IVF Width—Extrapolated Changes During Distraction

**Figure 6:**
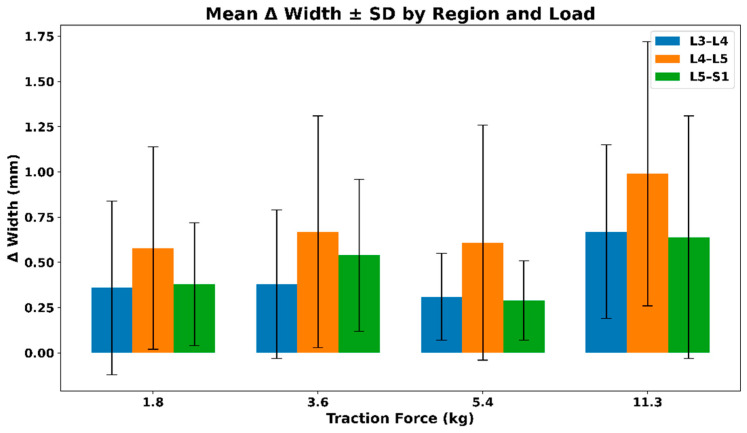
Mean ± SD change in intervertebral foramen (IVF) width across lumbar regions under graded distraction loads.

Figure 6 shows the mean ± SD change in intervertebral foramen width across lumbar regions under increasing distraction loads. All regions demonstrate positive mean width changes, with the largest absolute increases observed at higher loads, particularly at L4–L5. Variability increases with load, reflecting inter-subject differences in structural response rather than a uniform dose–response pattern. The mean ± SD values of the extrapolated intervertebral foramen (IVF) width across different traction loads are summarized in Table 3. 

#### 3.2.3. IVF Height—Extrapolated Changes During Distraction

**Figure 7:**
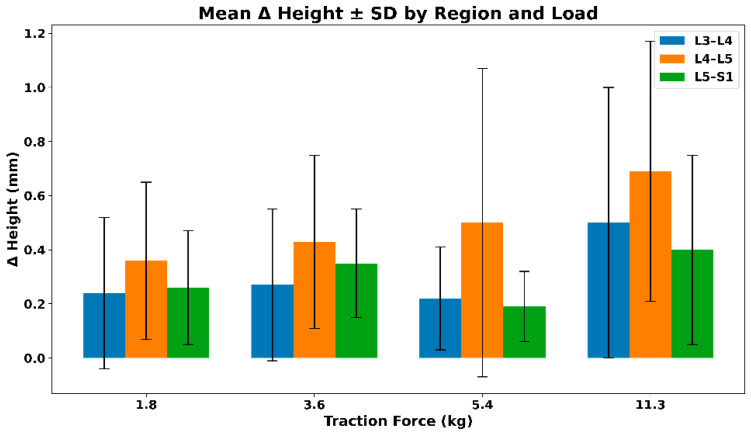
Mean (SD) change in intervertebral foramen (IVF) height across lumbar regions under graded distraction loads.

Figure 7 illustrates the mean (SD) change in intervertebral foramen height across lumbar regions under graded distraction loads. All regions demonstrate positive mean height changes, with the largest absolute increases observed at higher loads, particularly at L4–L5. Increasing variability with load reflects inter-subject differences in biomechanical response rather than a uniform dose–response relationship. The mean (SD) values of the extrapolated intervertebral foramen (IVF) height across different traction loads are summarized in Table 4.

#### 3.2.4. IVF Posterior Disc Height (PDH)—Extrapolated Changes During Distraction

**Figure 8:**
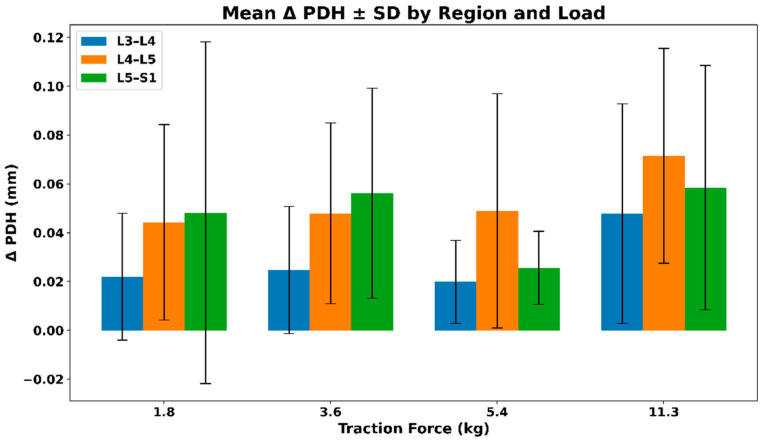
Mean (SD) change in posterior disc height (PDH) across lumbar regions under graded distraction loads.

Figure 8 presents the mean (SD) change in posterior disc height (PDH) across lumbar regions under graded distraction loads. All regions exhibit positive mean PDH changes, with larger absolute increases observed at higher loads, particularly at L4–L5 and L5–S1. Variability increases with load, reflecting inter-subject differences in disc response rather than a uniform load-dependent pattern. The mean (SD) values of the extrapolated posterior disc height (PDH) across different traction loads are summarized in Table 5.

#### 3.2.5. IVF Anterior Disc Height (ADH)—Extrapolated Changes During Distraction

**Figure 9:**
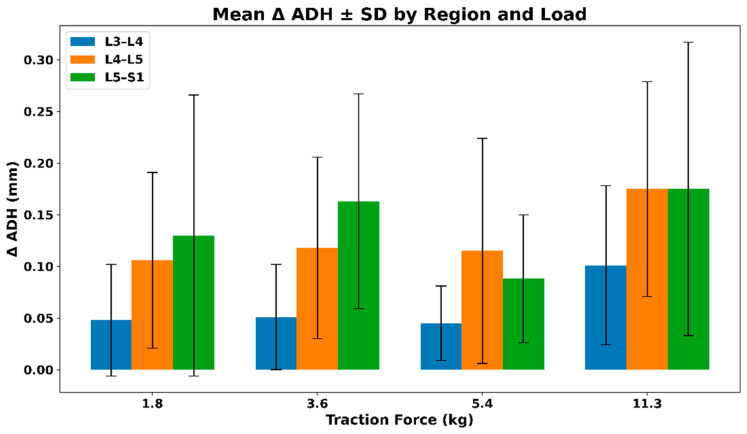
Mean (SD) change in anterior disc height (ADH) across lumbar regions under graded distraction loads.

Figure 9 illustrates the mean (SD) change in anterior disc height (ADH) across lumbar regions under graded distraction loads. All regions demonstrate positive mean ADH changes, with larger absolute increases observed at higher loads, particularly at L4–L5 and L5–S1. Variability increases with load, reflecting inter-subject differences in disc response rather than a uniform load-dependent pattern. The mean (SD) values of the extrapolated anterior disc height (ADH) across different traction loads are summarized in Table 6.

### 3.3. Analysis of the Extrapolated Data Set Conducted by the Generative AI Model

#### 3.3.1. Purpose of Generative AI Use and Bias Controls

Generative AI was introduced to provide a consistent and reproducible approach for interpreting complex biomechanical result tables, reducing variability associated with manual narrative reporting. The large language model (LLM) was used strictly as a post-analysis interpretive layer operating only on finalized, aggregated numerical values and was not involved in image annotation, scaling, extrapolation, statistical computation, or validation.

The LLM prompt was designed to enforce a non-clinical, descriptive scope, requiring neutral biomechanical language, exclusion of demographic or sensitive attributes, and avoidance of diagnostic, therapeutic, or causal claims. Bias controls and configuration constraints were aligned with FDA Good Machine Learning Practices (GMLP) and the NIST AI Risk Management Framework to ensure ethically constrained and bias-aware interpretation.

#### 3.3.2. Summary of LLM-Generated Interpretations

Based on aggregated pre-distraction baseline measurements and extrapolated distraction responses, the LLM consistently described region-dependent lumbar geometry and load-dependent deformation behavior across the studied spinal levels. At baseline, the upper lumbar regions (L3–L4 and L4–L5) exhibited larger mean values for intervertebral foramen area, height, and width, whereas the lumbosacral level (L5–S1) demonstrated smaller absolute dimensions. In contrast, disc height measures—anterior disc height (ADH) and posterior disc height (PDH)—were interpreted as comparatively more uniform across regions, suggesting that foraminal geometry contributes more strongly to inter-regional structural variation.

During distraction, the LLM reported positive delta changes across all measured variables, indicating expansion of lumbar structures under applied traction. The largest absolute responses were consistently observed at the highest applied load (25 lbs ≈ 11.3 kg). Among regions, L4–L5 demonstrated the greatest absolute increases in foraminal area and height, whereas L5–S1 showed comparatively larger proportional responses when normalized to its smaller baseline geometry. Responses across intermediate loads (4–12 lbs) were described as non-monotonic, reflecting heterogeneous biomechanical behavior rather than a simple linear dose–response relationship.

Across variables, the interpretation noted that variability increased with applied load, with standard deviations approaching or exceeding mean values in some conditions, highlighting substantial inter-subject differences in deformation magnitude. All observations were framed strictly as descriptive biomechanical patterns derived from aggregated data and do not imply clinical effectiveness, therapeutic benefit, or outcome relevance.

#### 3.3.3. Bias Validation Using the MicroEAI Ethical and Compliance Audit Platform

To independently evaluate bias and ethical compliance of the LLM-generated interpretations, a post hoc bias audit was conducted using the MicroEAI TSC+ platform. The evaluation analyzed the complete LLM interaction artifacts, including the interpretation prompt, model metadata, and generated output, under both Compliance and Audit evaluation modes.

**Figure 10:**
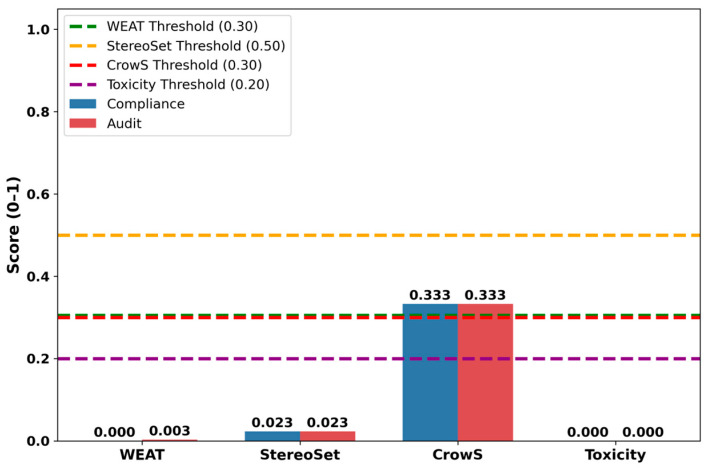
Bias detection metrics and threshold comparisons using WEAT, StereoSet, CrowS-Pairs, and toxicity analysis.

Bias (Figure 10) was quantified using established metrics, including the Word Embedding Association Test (WEAT), StereoSet, CrowS-Pairs, and toxicity analysis (Table 7). WEAT and toxicity scores were 0.000, indicating no detectable association bias or harmful language. The StereoSet score (0.023) reflected minimal stereotyping tendencies and remained well below predefined thresholds. The CrowS-Pairs score (0.333) marginally exceeded the nominal threshold; however, this signal was fully resolved through policy-defined whitelisting and human-in-the-loop review, resulting in no residual bias items requiring remediation.

The bias detection metric scores along with their corresponding evaluation thresholds are summarized in Table 7.

Although the CrowS-Pairs score marginally exceeds the nominal threshold, it remained within acceptable limits under both Compliance and Audit evaluation modes following policy-defined whitelisting and human-in-the-loop review, resulting in an overall approved bias verdict.

Based on the aggregated audit results, the LLM-generated interpretations were classified as low risk, with an overall Approved verdict under both evaluation modes. These findings confirm that the AI-generated text did not introduce measurable bias related to sensitive attributes and is suitable for inclusion as a non-clinical, descriptive interpretive aid in a biomechanical research context.

#### 3.3.4. AI Usage Disclosure

Generative AI was used in this study solely to assist in the interpretation of complex results derived from finalized numerical outputs of the biomechanical analysis. The AI system did not participate in image annotation, measurement extraction, geometric scaling, extrapolation procedures, or statistical computations. All analytical steps and numerical calculations were performed using deterministic computational methods, and the resulting AI-assisted interpretations were reviewed and verified by the authors prior to inclusion in the manuscript.

## 4. Discussion

In this study, we developed a methodology that integrates geometric scaling and extrapolation techniques to align spinal metrics between X-ray and ultrasound imaging. The computed scaling factor enabled standardized measurements, and extrapolation effectively aligned ultrasound-derived data with X-ray baseline values. Application of this methodology to fifteen volunteers demonstrated changes in intervertebral foramen (IVF) dimensions and disc heights under increased traction forces, consistent with previous research reporting that flexion–distraction procedures induce vertebral motion and increase IVF dimensions at the L4–L5 and L5–S1 levels in cadaver studies [7].

### 4.1. Clinical Significance and Imaging Modalities

Advanced imaging techniques provide quantitative and reliable spinal measurements, but each modality has its limitations. X-ray imaging, while rapid and widely available, emits ionizing radiation and has moderate reliability in measuring intervertebral disc height [5]. MRI, though highly detailed, is expensive and less accessible, whereas ultrasound imaging is non-invasive, cost-effective, and has been shown to yield reliable measurements comparable to MRI [16,17].

However, ultrasound imaging is operator-dependent, requiring precise probe positioning and expert interpretation. Studies evaluating ultrasound’s reliability in measuring lumbar interspinous distances found that inter-rater reliability declined over time, though the technique remained a useful tool for assessing lumbar segmental mobility [18]. Similarly, ultrasound demonstrated high intra- and inter-rater reliability in measuring deep cervical muscle dimensions in patients with cervical disc herniation, making it valuable for assessing muscle function and monitoring procedure interventions [19].

One challenge with ultrasound imaging is the time-consuming nature of manual annotations, which require clinical expertise. Ongoing research explores AI’s role in reducing reliance on manual labeling and improving ultrasound image interpretation efficiency [20,21,22,23,24,25,26].

### 4.2. Biomechanical Implications of the Study

This study primarily focused on analyzing foraminal and disc height changes under varying traction forces to quantify biomechanical spinal alterations. By demonstrating increased foraminal dimensions and disc height, we provide a quantitative framework for understanding how spinal manipulation and mobilization may contribute to clinical pain relief and functional improvement in future investigations. Increases in intervertebral foramen (IVF) dimensions are generally consistent with findings reported in previous studies [5,6,7].

### 4.3. Future Directions: Statistical Validation

While the present study incorporates an expanded cohort of fifteen healthy volunteers, the primary objective remains methodological validation and biomechanical characterization rather than inferential statistical testing. Accordingly, the current analysis emphasizes descriptive statistics and standardized extrapolation to establish reproducible deformation patterns across lumbar regions and traction loads.

Future studies with larger and more diverse cohorts, including participants with low back pain and specific spinal pathologies, will enable formal statistical validation. Planned analyses will include:

- Paired t-tests or Wilcoxon signed-rank tests to evaluate within-subject differences between pre-distraction and during-distraction measurements across traction loads.
- Repeated-measures ANOVA or mixed-effects models to assess force-dependent and region-specific variations in intervertebral foramen and disc-related metrics.
- Confidence intervals (CIs) to quantify uncertainty and precision of estimated biomechanical changes.
- Correlation and regression analyses to examine the relationship between applied traction forces and structural deformation across lumbar regions.

The incorporation of these statistical methods will support hypothesis-driven evaluation of traction-induced biomechanical effects and strengthen the translational relevance of the proposed imaging and extrapolation framework. Together with the standardized methodology established in this study, such analyses will contribute to a more robust evidence base for understanding lumbar spine mechanics under controlled distraction.

### 4.4. Integration of X-Ray and Ultrasound Imaging for Standardized Analysis

Recent advances in AI-based analysis of medical images have primarily focused on single imaging modalities, such as X-ray or ultrasound [27,28]. However, our methodology uniquely combines X-ray and ultrasound imaging to standardize spinal measurements across modalities, addressing a significant research gap.

The study’s main findings demonstrate that the use of a common scaling factor enabled standardized measurement across imaging modalities, allowing ultrasound-derived spinal metrics to be consistently aligned with X-ray–defined anatomical baselines. Analysis of data from fifteen healthy volunteers demonstrated reproducible changes in intervertebral foramen (IVF) dimensions and disc height measures under increased traction forces. These findings are consistent with prior reports of vertebral mobility and foraminal expansion during spinal decompression and flexion–distraction procedures.

## 5. Limitations

This study has several limitations that should be considered when interpreting the results. Although the cohort consisted of fifteen healthy adult volunteers, all participants were asymptomatic and did not have low back pain (LBP), which limits the generalizability of the findings to clinical populations with spinal pathology.

Ultrasound imaging was performed by a single registered sonographer, with one measurement acquired at the start of the procedure and one measurement acquired during each level of applied distraction. Similarly, X-ray imaging was performed by a single radiology technician, with baseline (pre-distraction) images acquired in a neutral position only. No X-ray imaging was performed during or after the distraction procedure, as X-rays were used solely to establish static anatomical baseline measurements. Consequently, inter-operator reliability and intra-operator repeatability could not be formally assessed.

While the current sample size supports descriptive biomechanical analysis and methodological validation, it was not designed for inferential statistical testing or outcome-based clinical conclusions. Future studies should include larger and more diverse cohorts, incorporating patients with LBP and specific spinal conditions such as disc degeneration, spinal stenosis, and spondylolisthesis (both true and pseudo). Such studies would enable correlation of biomechanical deformation patterns with clinical outcomes and symptom response.

In addition, larger-scale validation using automated or semi-automated annotation tools should be explored to improve efficiency, reduce observer dependency, and support scalability of the proposed framework.

## 6. Conclusions

This proof-of-concept study developed a standardized framework for aligning X-ray and ultrasound imaging using image annotation and deterministic mathematical scaling to derive a common geometric reference, enabling consistent alignment of static X-ray anatomy with dynamic ultrasound-derived deformation across graded traction loads. Using data from fifteen healthy adult volunteers, the study demonstrated reproducible, region-dependent changes in intervertebral foramen dimensions and disc height measures under traction, supporting the feasibility of combined X-ray–ultrasound assessment of lumbar biomechanics while minimizing repeated radiographic exposure.

The methodology enables systematic evaluation of spinal deformation and provides a foundation for analyzing mechanical responses under controlled distraction. Generative artificial intelligence was introduced to provide a structured and reproducible mechanism for translating finalized biomechanical results into consistent narrative interpretations, reducing manual interpretive variability while remaining strictly non-clinical and bias-controlled, and was not involved in measurement, scaling, extrapolation, or statistical computation.

Future work will extend this framework to larger and more diverse cohorts, incorporate formal statistical validation, relate biomechanical changes to clinical outcomes, and refine automated annotation techniques to support scalable multimodal spine research.

## Figures and Tables

**Table 1:** Pre-distraction mean ± SD values by lumbar region.

Region	ADH (mm)	Area (mm^2^)	Height (mm)	PDH (mm)	Width (mm)
L3–L4	1.62 (0.21)	58.89 (13.70)	7.73 (1.63)	0.73 (0.17)	11.65 (2.09)
L4–L5	1.62 (0.21)	44.38 (15.83)	6.91 (2.04)	0.76 (0.16)	9.85 (3.09)
L5–S1	1.87 (0.54)	17.67 (8.79)	4.17 (1.26)	0.60 (0.24)	6.39(2.44)

**Table 2:** Mean ± SD values of extrapolated intervertebral foramen (IVF) area across traction loads.

Force (Kgs)	L3–L4	L4–L5	L5–S1
1.8	3.93 (5.56)	5.32 (6.01)	2.08 (1.88)
3.6	4.27 (4.89)	6.25 (6.05)	3.18 (2.67)
5.4	3.28 (2.81)	6.36 (7.30)	1.63 (1.27)
11.3	7.27 (6.34)	9.94 (8.02)	4.09 (4.88)

**Table 3:** Mean ± SD values of extrapolated intervertebral foramen (IVF) width across traction loads.

Force (kg)	L3–L4	L4–L5	L5–S1
1.8	0.36 (0.48)	0.58 (0.56)	0.38 (0.34)
3.6	0.38 (0.41)	0.67 (0.64)	0.54 (0.42)
5.4	0.31 (0.24)	0.61 (0.65)	0.29 (0.22)
11.3	0.67 (0.48)	0.99 (0.73)	0.64 (0.67)

**Table 4:** Mean (SD) values of extrapolated intervertebral foramen (IVF) height across traction loads.

Force (kg)	L3–L4	L4–L5	L5–S1
1.8	0.24 (0.28)	0.36 (0.29)	0.26 (0.21)
3.6	0.27 (0.28)	0.43 (0.32)	0.35 (0.20)
5.4	0.22 (0.19)	0.50 (0.57)	0.19 (0.13)
11.3	0.50 (0.50)	0.69 (0.48)	0.40 (0.35)

**Table 5:** Mean (SD) values of extrapolated posterior disc height (PDH) across traction loads.

Force (kg)	L3–L4	L4–L5	L5–S1
1.8	0.02 (0.026)	0.04 (0.040)	0.04 (0.070)
3.6	0.02 (0.026)	0.04 (0.037)	0.05 (0.043)
5.4	0.01 (0.017)	0.04 (0.048)	0.02 (0.015)
11.3	0.04 (0.045)	0.07 (0.044)	0.05 (0.050)

**Table 6:** Mean (SD) values of extrapolated anterior disc height (ADH) across traction loads.

Force (kg)	L3–L4	L4–L5	L5–S1
1.8	0.04 (0.054)	0.10 (0.085)	0.13 (0.136)
3.6	0.05 (0.051)	0.11 (0.088)	0.16 (0.104)
5.4	0.04 (0.036)	0.11 (0.109)	0.08 (0.062)
11.3	0.10 (0.077)	0.17 (0.104)	0.17 (0.142)

**Table 7:** Bias detection metric scores and corresponding evaluation thresholds.

Bias Metric	Compliance Score	Audit Score	Threshold	Status
WEAT	0	0.003	≤0.30	Within threshold
StereoSet	0.023	0.023	≤0.50	Within threshold
CrowS-Pairs	0.333	0.333	≤0.30	Slightly above threshold *
Toxicity	0	0	≤0.20	Within threshold

* CrowS-Pairs score marginally exceeds the nominal threshold (0.30) but remains within acceptable limits.

## Data Availability

The data for the project is available upon request.

## Supplementary Materials

The following supporting information can be downloaded at: https://www.mdpi.com/article/10.3390/bioengineering13030330/s1, Table S1: Demographic Data.

## Abbreviations

The following abbreviations are used in this manuscript:

ADH	Anterior disc height
AI	Artificial intelligence
CSF	Common scaling factor
CT	Computed tomography
GenAI	Generative AIUS
HSF	Horizontal scaling factor
IVF	Intervertebral foramen
LBP	Low back pain
MRI	Magnetic resonance imaging
PDH	Posterior disc height
VSF	Vertical Scaling Factor

## Appendix A

### Appendix A.1. Calculations and Scaling Factor Formulas

The approach relies heavily on mathematical computations to ensure standardized measurements across imaging modalities. Key formulas and their applications are detailed below:

**Formula** **A1:**
*IVF Area Calculation (Shoelace Theorem).*

*The polygon area formula used to calculate the IVF area from the annotation data is based on the Shoelace Theorem (Gauss’s area formula) for a simple closed polygon. For a given set of nnn coordinate points (x1, y1), (x2, y2), …, (xn, yn)(x_1, y_1), (x_2, y_2), …, (x_n, y_n)(x1, y1),(x2, y2), …, (xn, yn) representing the boundaries of the IVF region:*

$$Area=\frac{1}{2}\left|\sum_{i=1}^{n-1}\left(x\_i\;y\_(i+1)-x\_(i+1)\;y\_i\right)+(x\_n\;y\_1-x\_1\;y\_n)\right|$$

**Formula** **A2:**
*Distance Calculation Formula:*

$$Distance=\sqrt{\left\{{\left(x_{2}-x_{1}\right)}^{2}+{\left(y_{2}-y_{1}\right)}^{2}\right\}}$$

*Purpose: Used to calculate horizontal and vertical lumbar distances from annotated points on X-ray and ultrasound images.*

**Formula** **A3:** Conversion of Pixel Measurements to Real-World Units (mm or cm):

$$Real-World\;Distance=\frac{\left\{Pixel\;Distance\times 25.4\right\}}{\left\{DPI\right\}}$$

*Explanation: This formula converts pixel measurements obtained from annotations into millimeters or centimeters using the DPI of the images, accurately representing the physical distances of the annotated points.*

Scaling Factor Calculations:

Where:

- CSF = Common Scaling Factor
- GMean = Geometric Mean function

This formula ensures a unified scaling adjustment by averaging both horizontal and vertical scaling factors, enabling consistent measurement standardization across imaging modalities.

**Formula** **A4:** *Area Calculations*:

Area=Width∗Height∗Scaling Factor

*Explanation: Annotated width and height values were multiplied and adjusted using the scaling factors to accurately estimate the area of the intervertebral foramen (IVF) or other anatomical structures.*

**Formula** **A5:**
*Adjustments for Disc Heights (PDH and ADH):*

*Adjusted Height* = *Annotated Height* × *Vertical Scaling Factor*

*Explanation: PDH (Posterior Disc Height) and ADH (Anterior Disc Height) values were adjusted using the vertical scaling factor to ensure alignment with real-world measurements.*

### Appendix A.2. Image Acquisition and Parameters

X-ray Imaging:

- Dimensions: 3008 × 3008 pixels
- Horizontal and Vertical Resolution: 96 dpi
- Bit Depth: 24

Ultrasound Imaging:

- Dimensions: 1552 × 970 pixels
- Horizontal and Vertical Resolution: 96 dpi
- Bit Depth: 24

**Formula** **A6:**
*Extrapolation Formula:*

*Adjusts ultrasound measurements to match X-ray scales using calculated scaling factors.*

$$Extrapolated\;Value=X-Ray\;Base*\left(\frac{US\;Pixel\;Value}{dpi}*25.4\right)*Scaling\;Factor$$
